# Anti-proliferative and cytotoxic effect of cannabidiol on human cancer cell lines in presence of serum

**DOI:** 10.1186/s13104-020-05229-5

**Published:** 2020-08-20

**Authors:** Alberto Sainz-Cort, Claudia Müller-Sánchez, Enric Espel

**Affiliations:** 1 GH Medical, Barcelona, Spain; 2 Celltec-UB, Department of Cell Biology, Physiology and Immunology, Faculty of Biology, University of Barcelona, Av. Diagonal 643, 08028 Barcelona, Spain

**Keywords:** Paclitaxel, Colon cancer, Cannabidiol, Serum

## Abstract

**Objective:**

Cannabinoids are able to reduce tumor growth in xenograft models, but their therapeutic potential as anti-cancer drugs in humans is unclear yet. In vitro studies of the effect of cannabinoids on cancer cells are often carried out in absence of serum or in low serum concentration (i.e. 0.5% serum), conditions that limit cellular growth and therefore can increase the response of cells to additional challenges such as the presence of cannabinoids. However, the tumor microenvironment can be teaming with growth factors. In this study we assessed the viability and proliferation of cancer cells treated with cannabidiol in presence of a serum concentration that commonly sustains cell growth (10% serum).

**Results:**

The results show that cannabidiol exerts a markedly different effect on the viability of the human HT-29 cancer cell line when cultured in presence of 0.5% serum in comparison to 10% serum, displaying a cytotoxic effect only in the former situation. In presence of 10% serum, no inhibitory effect of cannabidiol on DNA replication of HT-29 cells was detected, and a weak inhibition was observed for other cancer cell lines. These results indicate that the effect of cannabidiol is cell context-dependent being modulated by the presence of growth factors.

## Introduction

The cannabis plant has a therapeutic potential to treat a wide range of diseases, including cancer. Phytocannabinoids, are being tested in vitro and in vivo for the potential to fight different types of cancer. Cannabis extracts have recently been described to exert a cytotoxic effect on human cancer cell lines [13].

However, in vitro cancer models, present limitations which reduce their predictive validity. One of these limitations is to reproduce the nutritional environment of the cells using cell culture media and growth factors [1]. Many in vitro cancer studies use historical culture media with fetal calf serum (FCS). However, it is usual to eliminate or reduce FCS concentrations (i.e. FCS < 5%) from the media at the moment of drug exposure to avoid confounding effects of growth factors present in serum, as in many studies testing the cytotoxic properties of cannabinoids in cancer cells [12, 14, 15].

The deprivation of survival factors from the media can sensitize cells to a subsequent challenge. Pirkmajer and Chibalin [10] showed that the effects of serum starvation in cell cultures are unpredictable. According to Eastman [3], serum should be kept in cell cultures to avoid both false positive and negative results due to its effects on cell proliferation, stipulating the importance of replicating organic conditions to obtain clinically valid results.

In the present study, we analyzed the viability response of different cancer cell lines to cannabidiol (CBD) in presence of a standard concentration of serum (10%) in comparison to a low serum concentration (0.5%).

## Main text

### Materials and methods

#### Materials

CBD was supplied by Schibano Pharma AG (Wald*-*Schönengrund, Switzerland). McCoy’s 5A medium, Leibovitz's *L*-*15* medium (L-15) and RPMI 1640 and AlamarBlue*®* (AB) (Invitrogen) were bought from ThermoFisher Scientific (Barcelona, Spain). Paclitaxel, 4′,6-diamidino-2-phenylindole (DAPI), dimethyl sulfoxide, L-glutamine, penicillin–streptomycin and FCS were bought from Sigma-Aldrich (Madrid, Spain). Cell Proliferation Reagent WST-1 and 5-bromo-2′-deoxyuridine (BrdU) cell proliferation Elisa kit were bought from Roche, Sigma-Aldrich (Madrid, Spain). Paclitaxel was dissolved in dimethyl sulfoxide and CBD was dissolved in methanol at 80 mM and kept at −80 °C for a maximum of 2 months. When needed, Paclitaxel and CBD were diluted conveniently in the cell media at the indicated final concentrations. Cellular controls without CBD or Paclitaxel contained cell media without additives.

#### Cell culture

HT29 cells (ref. HTB-38) and SW480 cells (ref. CCL-228) were obtained from American Type Culture Collection. AGS cells were kindly provided by Miguel A. Pujana (Catalan Institute of Oncology, IDIBELL, Barcelona, Spain) and were originally obtained from Nuria Sala (Catalan Institute of Oncology, IDIBELL, Barcelona, Spain). Human colon cancer HT-29 cells and SW480 cells were maintained in McCoy’s 5A and L-15 media, respectively. Human gastric cancer AGS cells, kindly provided by Francesca Mateo (Catalan Institute of Oncology, Bellvitge Institute for Biomedical Research, L’Hospitalet del Llobregat, Spain) were maintained in RPMI medium. All of the media was supplemented with 1% penicillin–streptomycin and 2 nM L-Glutamine. 24 h before treatment, cells were plated in 96-well plates at 500–1000 cells/well. 24 h later, wells in triplicates received CBD and Paclitaxel. All assays with SW480 and AGS cells included 10% FCS, while the assays using HT-29 cells included either 10 or 0.5% FCS.

#### Cell viability and proliferation assays

For the viability and proliferation assay based on resazurin and its redox-mediated reduction we used 10% AB and measured the fluorescence of the wells using a plate reader.

For the viability and proliferation assay based on cleavage of tetrazolium salts by mitochondrial dehydrogenase we used 10% WST-1.

For the proliferation based on the measurement of DNA synthesis we added BrdU to cells and detected its incorporation into DNA following manufacturer instructions.

To assess cell viability, DAPI was added to the cell suspension 5 min before the analysis by flow cytometry. DAPI, emits higher fluorescence when bound to DNA. DAPI enters rapidly through altered cell membranes allowing the detection of damaged cells. The cell population was selected by gating in a forward scatter vs. side scatter dot plot, excluding aggregates and cell debris. Samples were analyzed using a Gallios flow cytometer.

#### Statistical analysis

Data was analysed using IBM SPSS Statistics 23 and Real Statistics Using Excel.

We used Shapiro–Wilk test to assess data normality and non-parametrical independent samples Kruskal–Wallis test to identify significant differences between each experimental condition. We used Dunn test as a post-hoc analysis to identify which groups show statistically significant differences.

### Results

#### Viability and proliferation of HT-29 cells with serum deprivation (0.5% FCS)

**Fig. 1:**
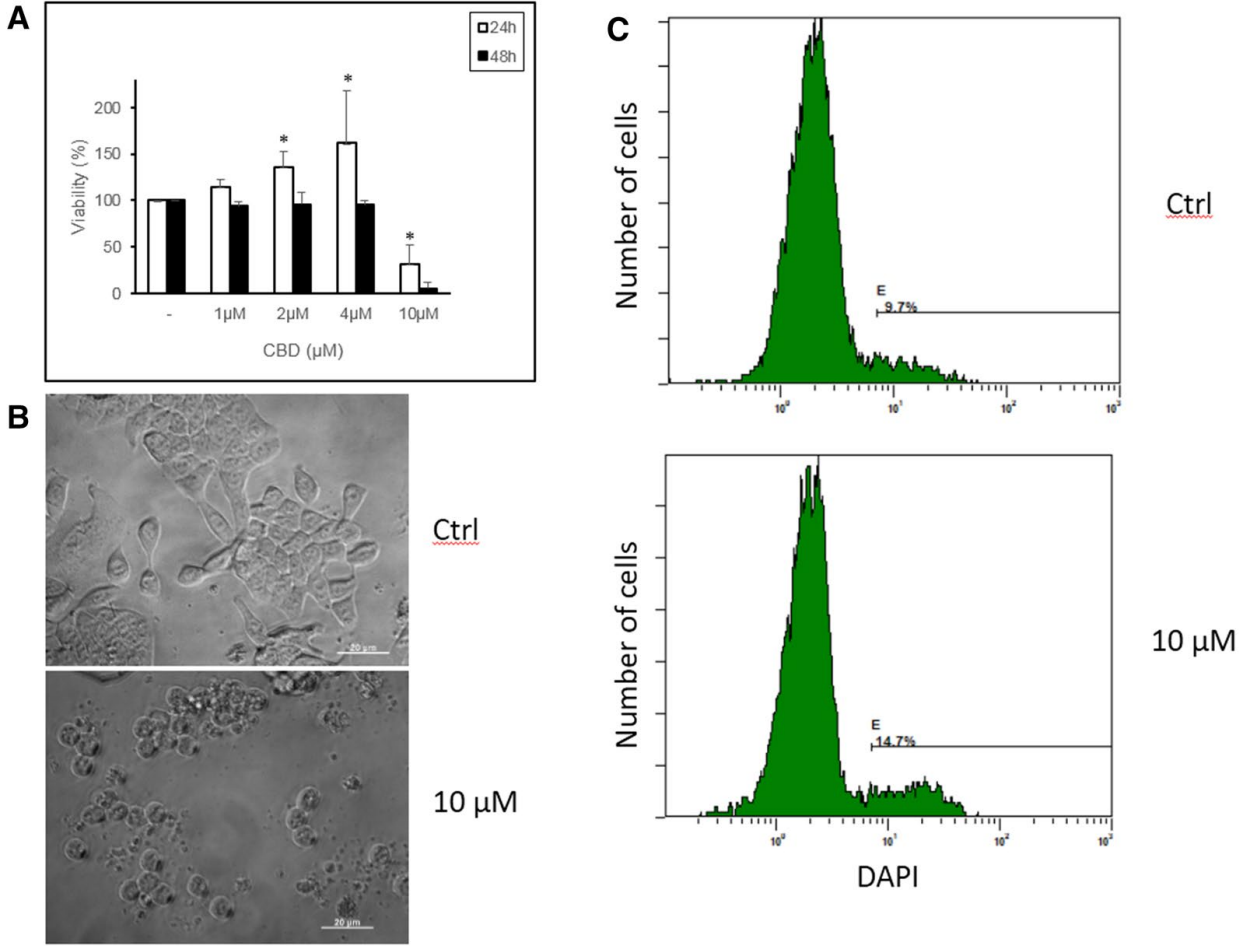
**a** HT-29 cells were incubated with 0.5% FCS and different concentrations of CBD for 24 and 48 h. Cell viability was assessed by incubation with AB. The mean + SD of three assays are shown. **b** Morphology of HT-29 cells incubated with or without 10 μM CBD for 24 h. Representative images are shown (bar, 20 µm). **c** HT-29 cell viability according to DAPI staining (see the “Materials and methods” section). HT-29 cells were incubated without (top) or with 10 μM CBD (bottom) for 24 h, stained with DAPI and immediately analyzed by flow cytometry. The cursor identifies DAPI-positive cells (dead cells), showing a higher percentage in CBD-treated cells. A representative experiment (**c**) is shown. *p < 0.05

When human colon cancer HT-29 cells were incubated in media with 0.5% serum, adding CBD at 10 µM reduced cell viability as assessed via the resazurin method, which is based on evaluating mitochondrial reductive capacity [11] (Fig. 1a). Interestingly, when CBD concentrations were ≤ 4 µM, cell viability increased during the first 24 h. Differences between 2 or 4 and 10 µM were statistically significant (p = 0.006 and p = 0.013). At 48 h, the increasing viability with CBD ≤ 4 µM disappeared while the blocking effect of 10 µM CBD was more pronounced (Fig. 1a). This suggests that CBD can induce mitochondrial stress, as reported by others [18]. Looking at the morphology of cells, the treatment with 10 µM CBD led to changes in cell form, such as massive cellular detachment, cell rounding and presence of wrinkled cells characteristic of dead cells (Fig. 1b). In fact, analyzing the presence of dead cells using DAPI dye, we found an increased percentage in samples incubated with 10 µM CBD when compared to control cells (Fig. 1c). Thus, the loss of mitochondrial activity observed at CBD 10 µM correlated with cell death. Of note, at longer incubation times (i.e. 5 days) massive cellular death was also observable at 4 µM CBD (data not shown). In summary, 10 µM CBD shows cytotoxic activity on HT-29 cells cultured in 0.5% FCS.

#### Viability and proliferation of HT-29 cells in 10% FCS

**Fig. 2:**
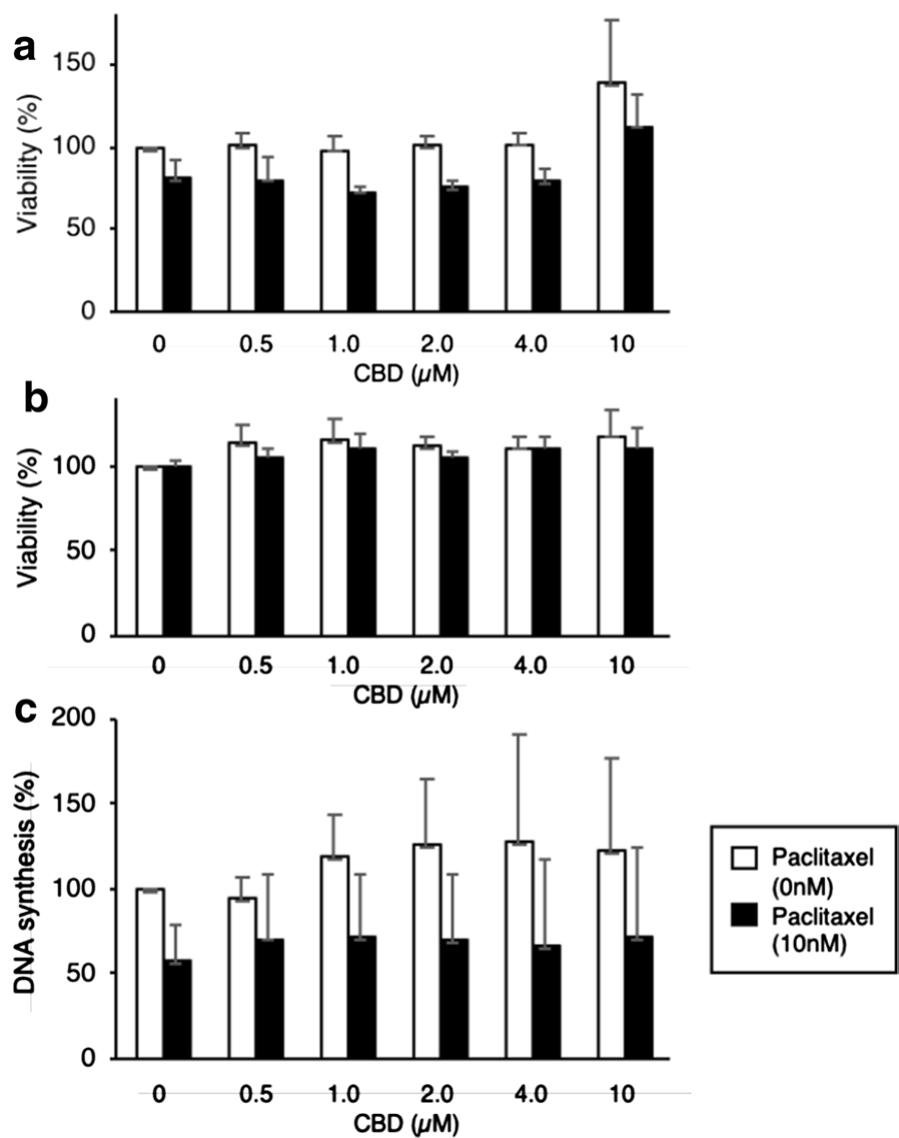
HT-29 cells were incubated for 3 days with 10% FCS and different concentrations of CBD in absence or presence of 10 nM paclitaxel. **a** The viability was assessed by incubation with AB. The mean + SD are shown (n = 3). **b** The viability was assessed by incubation with WST-1. The mean + SD are shown (n = 3). **c** Before harvesting, cells were incubated with BrdU for 2 h, which incorporated into DNA, and DNA synthesis was quantified. The mean + SD are indicated (n = 3)

Contrary to the drop in viability of cells in 0.5% FCS, CBD did not inhibit the viability of HT-29 cells even after 3 days in media containing 10% FCS (Fig. 2a, b). An apparent increase in HT-29 cell viability was observed at 10 µM CBD, as assessed by AB or WST-1 (Fig. 2), suggesting mitochondrial stress. We sought to find whether in these conditions CBD could show additive or synergistic anti-proliferative effects with the therapeutic drug paclitaxel. Paclitaxel partially decreased the viability of HT-29 cells, according to AB measurement, but not WST-1. Thus, CBD at 10 µM does not grossly affect the viability of HT-29 cells after 3 days culture in presence of 10% serum.

To ascertain whether CBD had any effect on proliferation of HT-29 cells we measured the incorporation of BrdU into DNA. No changes in DNA synthesis were observed after 3 days of incubation of HT-29 cells with any concentration of CBD (Fig. 2c). Although paclitaxel in itself did inhibit DNA synthesis, CBD did not increase the effect of Paclitaxel (Fig. 2c). In summary, CBD up to 10 µM do not decrease the viability nor the proliferation of HT-29 cells cultured in 10% FCS. None of these results showed statistically significant differences.

#### Viability and proliferation of SW480 and AGS cells

To know whether other cancer cell lines behaved similarly to HT-29, showing little or no response to CBD when cultured in 10% FCS we used SW480, another colon cancer cell line and AGS, a gastric cancer cell line.

**Fig. 3:**
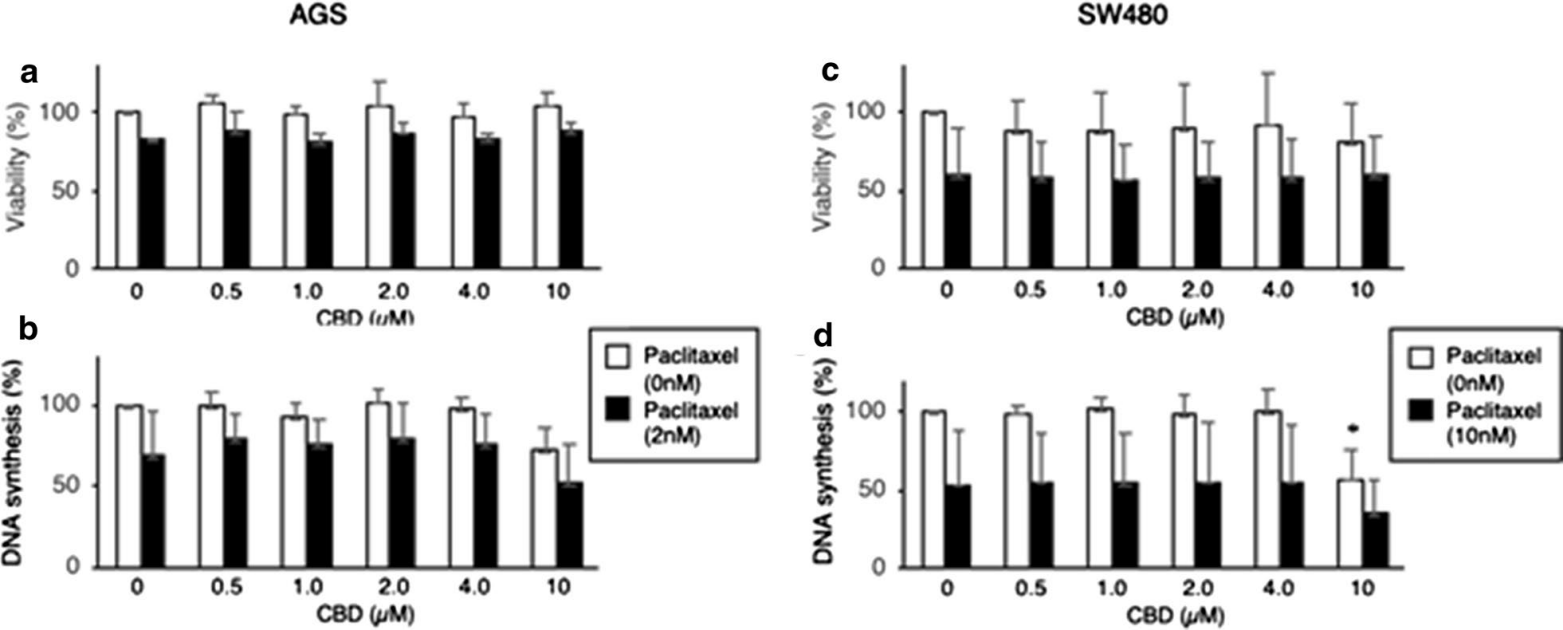
AGS cells and SW480 cells were incubated for 3 days with different concentrations of CBD in absence or presence of 2 nM Paclitaxel (AGS) or 10 nM Paclitaxel (SW480). **a**, **c** Cell viability was assessed by incubation with AB. The mean + SD of three (AGS) and six (SW480) assays are shown. **b**, **d** Before harvesting, cells were incubated for 2 h with BrdU, which incorporated into DNA, and DNA synthesis was quantitated. The mean + SD of three assays (AGS) and 5 assays (SW480) are shown. *p < 0.05

AGS cells did not show changes of viability by incubation with CBD up to 10 µM, though 2 nM Paclitaxel did decrease their viability (Fig. 3a). Higher Paclitaxel concentrations resulted in a severe decrease of AGS cells viability (data not shown) so we used 2 nm Paclitaxel to observe potential effects of CBD. The viability of SW480 cells with CBD and 10% FCS showed a trend to decline (Fig. 3c). Surprisingly and contrary to HT-29 cells, 10 µM CBD did actually impair DNA replication in AGS and SW480 cells (Fig. 3b, d). In fact, the inhibition of DNA replication was additive to that produced by Paclitaxel. The assessment of DNA replication in SW480 cells showed significant differences between the control sample and 10 µM CBD without paclitaxel (p = 0.021). Any other statistic analysis did not show significant results.

In summary, in presence of 10% FCS and during 3 days of culture, CBD does not affect the viability of HT-29, SW480 and AGS cells, though CBD at 10 µM does impair the proliferation of AGS and SW480 cells.

### Discussion

In this study, we investigated the effects of CBD and its combination with Paclitaxel on the viability of three different cancer cells (HT-29, SW480 and AGS) under two different concentrations of serum, a standard 10% appropriate for cell growth (for HT-29, SW480 and AGS) and a restrictive one of 0.5% (for HT-29 only). For HT-29 cells, CBD only reduces cell viability under low FCS, with no effects on viability or DNA replication when cells were in 10% FCS. However, for SW480 and AGS, DNA replication was impaired under 10 µM CBD with 10% serum. Moreover, the inhibition of DNA replication in SW480 and AGS cells by CBD and Paclitaxel had an additive effect.

At low CBD concentrations HT-29 cells showed a trend towards increased cell viability, though the differences were not significant. Different concentrations of CBD have previously been shown to have opposing effects on cells. Thus, 1 µM CBD induces proliferation of T leukemia cells, but at higher concentration kills the cells [9]. A low concentration, CBD increases mitochondrial Ca^2+^ augmenting mitochondrial metabolism and cell growth, but at high concentration, it leads to excessive mitochondrial Ca^2+^, mitochondrial dysfunction and cell death [9].

Appropriate culturing conditions are essential for the survival and growth of cells. In many studies, cell culture conditions are not sufficiently detailed, which is essential for study replication. One possible solution to address the potential effect of serum could be using culture media without FCS, so the media does not need to be altered during drug exposition [17]. In any case, neither higher serum concentrations nor lower serum concentrations represent the proper microenvironment of a cancer cell in the human body, and both approaches could be valid to test the effects of a drug on cell lines. The tumor microenvironment is enriched with metabolites including lactate and adenosine [2, 4], which increases tumor growth and may modulate the therapeutic effect of a drug. In tumors that are highly glycolytic, increasing mitochondrial activity as exerted by CBD, may add metabolic stress to cells forcing them to decreased growth [5]*.* The effect of a drug on cells can be assessed effectively if the experimental conditions of the treatment are the same as the growing conditions before the treatment. Once growing conditions and treatment conditions differ from more than one variable (drug treatment) then the resulting effects cannot be associated only to the treatment but to the combination of variables.

## Limitations

Our results did not show statistically significant differences with the exception of the assessment of viability of HT-29 cells under CBD treatment and the assessment of DNA replication of SW480 under 10 µM CBD. The lack of statistically significant results could be due to the small sample size (n = 3 for most of the assays). Our study was also not able to replicate the strongly inhibitory effect of CBD shown in other studies where cannabinoids were tested against cancer cells cultured with 10% FCS. FCS contains many growth factors and nutrients, and differences in the FCS source could substantially modify the viability, proliferation and differentiation of cultured cells. There are also other studies where cancer cells were cultured with 10% FCS and treated with CBD or other synthetic CBD-like molecules. The results of these studies showed that CBD (5–20 μg/mL) reduced the viability of cancer cells and also had effects on other survival variables [6–8, 16]. The cell lines used in these studies being different to the ones used in our study, could account for the different results observed.

## Data Availability

The datasets used and/or analyzed during the current study are available from the corresponding author on reasonable request.

## Abbreviations

AB: alamarBlue
BrdU: 5-bromo-2′-deoxyuridine
CBD: cannabidiol
DAPI: 4′,6-diamidino-2-phenylindole
FCS: fetal calf serum
